# Abnormal Pre-Attentive Arousal in Young Children with Autism Spectrum Disorder Contributes to Their Atypical Auditory Behavior: An ERP Study

**DOI:** 10.1371/journal.pone.0069100

**Published:** 2013-07-25

**Authors:** Tatiana A. Stroganova, Vladimir V. Kozunov, Irina N. Posikera, Ilia A. Galuta, Vitaliy V. Gratchev, Elena V. Orekhova

**Affiliations:** 1 The MEG Centre, Moscow State University of Psychology and Education, Moscow, Russia; 2 Laboratory of developmental psychogenetics, Psychological Institute of Russian Academy of Education, Moscow, Russia; 3 Clinical Department for the Study of Adolescent Psychiatry, Mental Health Research Center of Russian Academy of Medical Sciences, Moscow, Russia; 4 MedTech West, Sahlgrenska Academy, Gothenburg, Sweden; Tokyo Metropolitan Institute of Medical Science, Japan

## Abstract

Auditory sensory modulation difficulties and problems with automatic re-orienting to sound are well documented in autism spectrum disorders (ASD). Abnormal preattentive arousal processes may contribute to these deficits. In this study, we investigated components of the cortical auditory evoked potential (CAEP) reflecting preattentive arousal in children with ASD and typically developing (TD) children aged 3-8 years. Pairs of clicks (‘S1’ and ‘S2’) separated by a 1 sec S1-S2 interstimulus interval (ISI) and much longer (8-10 sec) S1-S1 ISIs were presented monaurally to either the left or right ear. In TD children, the P50, P100 and N1c CAEP components were strongly influenced by temporal novelty of clicks and were much greater in response to the S1 than the S2 click. Irrespective of the stimulation side, the ‘tangential’ P100 component was rightward lateralized in TD children, whereas the ‘radial’ N1c component had higher amplitude contralaterally to the stimulated ear. Compared to the TD children, children with ASD demonstrated 1) reduced amplitude of the P100 component under the condition of temporal novelty (S1) and 2) an attenuated P100 repetition suppression effect. The abnormalities were lateralized and depended on the presentation side. They were evident in the case of the left but not the right ear stimulation. The P100 abnormalities in ASD correlated with the degree of developmental delay and with the severity of auditory sensory modulation difficulties observed in early life. The results suggest that some rightward-lateralized brain networks that are crucially important for arousal and attention re-orienting are compromised in children with ASD and that this deficit contributes to sensory modulation difficulties and possibly even other behavioral deficits in ASD.

## Introduction

One striking feature of individuals with autism spectrum disorders (ASD) is the narrow, ‘spotlight’ attention and associated difficulties with attention shifting. While focusing on a stimulus or activity, people with autism demonstrate decreased awareness of social and non-social stimuli beyond the focus of their attention. Young children with autism are considerably slower to reorient to peripheral visual events than their typically developing (TD) peers when their attention is engaged by a central stimulus [1]. However, when events occur within the focus of their attention, individuals with autism may demonstrate superior sensory-perceptual abilities in both the auditory and visual domains [2,3].

The prolonged time needed for reorienting to peripheral visual stimuli in infant siblings of children with autism is associated with a later diagnosis of autism [4]. A similar deficit exists in the auditory domain and is especially striking during the first years of life. Many infants and toddlers with autism are so unresponsive to sound that parents suspect hearing loss [5]. On the other hand, hypersensitivity to sound, or hyperacusis, is also a very common problem in ASD, especially in early life [6]. Ben-Sasson et al. [7] reported remarkably frequent co-occurrence of auditory hyper- and hyposensitivity symptoms in children with ASD and suggested that both of these problems may be explained by a common mechanism, such as a dysfunctional arousal system, that compromises the ability to regulate an optimal response.

The neural origins of such abnormal behavioral responses in children with autism are poorly understood, and the causal link between these dysfunctions and attention abnormalities is unclear. Theoretically, problems with stimulus-driven reorienting may stem from dysfunction of the distributed cortical networks for controlling attention.

Corbetta et al. [8] have proposed that reorienting to biologically salient or task-relevant stimuli that appear outside the focus of attention is subserved by a ventral attention network, which includes the temporoparietal junction (TPJ) cortex and ventral frontal cortical areas predominantly of the right hemisphere [8,9]. These cortical epicenters are interconnected with each other and with subcortical structures involved in arousal regulation. Numerous clinical observations and experimental findings have indicated that damage to the cortical or subcortical components of this network or of their connections leads to impairment of attention reorienting, especially if the lesion is to the right side of the brain [10–12]. Given that the orienting network operates on multiple anatomical levels and time scales, EEG and MEG findings in autism are of special interest because they may help to reveal putative alternations, even in early preattentive processes, which may in turn affect later processing stages.

Event-related potential studies of involuntary orienting responses to salient changes in the acoustic environment in ASD used two main experimental paradigms.

The novelty ‘odd-ball’ paradigm was applied to investigate brain response to a unique novel sound embedded in a sequence of repetitive ‘non-target’ standard and target deviant stimuli. The anterior positive component of the cortical auditory evoked potential (CAEP) to novel stimuli with a latency of approximately 300 msec (A/Pcz/300) reflects involuntary orienting of attention to unexpected events [13,14]. Abnormal reduction of the A/Pcz/300 has been found in children with ASD [15,16], suggesting deficit at this rather late processing stage. These findings also raise the possibility of abnormalities at even earlier preattentive processing stages preceding orientation toward as yet unattended to but potentially significant sounds.

The ‘sensory gating’ paradigm is applied to investigate early preattentive stages of auditory processing. Pairs of clicks (‘S1’ and ‘S2’) separated by short within-pair interstimuli intervals (ISIs) are presented with much longer inter-pair ISIs. The so-called ‘obligatory’ components (P1 with a latency of 50-80 msec and N1 at approximately 100 msec) of the adult CAEP decreases in amplitude with repetition of stimuli with short ISIs (i.e., S2/S1 < 1). It was suggested that the larger response to S1 presented after a long interval of silence is due to higher bottom-up arousal and/or lower predictability of the auditory stimulus [17,18]. In this case, components’ amplitudes reflect early automatic allocation of resources for processing a temporally novel event, in other words, an arousal and initial orienting response [19]. This mechanism of orienting toward temporally or contextually novel sounds (‘gating-in’) is fundamentally different from another process that is triggered by repetition of the same sound with short time intervals and reflects inhibitory dampening (‘gating-out’) of repetitive irrelevant auditory stimuli [20,21]. While preattentive arousal toward novel sound is measured by the amplitudes of S1-related components, sensory ‘gating-out’ is usually defined as the S2/S1 amplitude ratio. Pronounced amplitude suppression in response to the second click corresponds to a robust inhibitory function of the brain, i.e., a normal sensory gating process [22]. Both decreased S1 amplitudes and increased S2/S1 ratios of P1 and N100 components were observed in some psychiatric disorders, such as schizophrenia [23–25]. The two types of abnormalities, however, may reflect different neurocognitive deficits [21] and can be differentially modulated by neuro-pharmacological agents [26].

Our recent study has shown that in children the vertex-positive deflection of potential between 50–130 msec (‘P1’) in response to clicks is characterized by presence of *two* distinct components: P50 (at approximately 65 ms) and P100 (at approximately 100 ms) [27]. The P100 is followed by and partly overlaps with the component N1c (also called Tb) that peaks at temporal sites with a latency of approximately 140 msec [28]. No amplitude abnormalities of the early P1 component (P50, i.e. at approximately 50 msec) were observed in children with autism in response to temporally novel S1 click [29–31]. The reduction of the S1 amplitude has been, however, found for the later obligatory component N1c [28], which in this study was right-lateralized in typically developing 4-8-year-old children, but was strongly reduced at the right side in 4-8-year-olds with autism. We interpreted this finding as evidence for abnormalities in networks for attention re-orienting [8,32,33] in ASD. In a recent MEG study of older children with autism, we analyzed the P100m component peaking at approximately 100 msec after the clicks. The P100 is the most prominent component of the auditory magnetic field response to infrequent clicks in children [34] and might reflect preattentive arousal abnormalities even earlier than N1c. In line with the N1c/EEG findings, this MEG study has shown that children with ASD lacked normal right-hemispheric predominance of the P100m, which has been observed in the TD group. Moreover, the P100m abnormalities in children with ASD correlated with auditory modulation difficulties, thus suggesting that they may contribute to abnormal sensory behavior in ASD.

Little is known about functional properties of the P100 component in children. In adults, on the other hand, P1(P50) with a latency of 50-80 msec has been related to a generator substrate within the cholinergic branch of the ascending reticular activating system (RAS) and its thalamic and cortical projections [35]. In the CNS, acetylcholine acts through two main types of receptors: nicotinic and muscarinic. Recent findings suggest that nicotine pathways are particularly strongly compromised in ASD [36]. The nicotine receptor-mediated transmission is critically involved in regulation of attention disengagement and shifting [37–39]. Its dysfunction may therefore contribute to both P1/P100 abnormalities and attention re-orienting problems in individuals with ASD. These considerations make the early arousal-related CAEP components especially interesting to study in autism.

Based on our previous research, we proposed that abnormal behavioral responses to auditory stimulation in children with ASD might reflect a deficiency of the preattentive arousal stages that critically depend on the right-lateralized brain networks and precede shifting of attention to physically salient and temporally novel stimuli.

To verify the hypothesis of lateralized brain deficits, it is important to study CAEP responses under the condition of lateralized stimulus presentation from the left and right hemi-spaces. Due to the crossing of the centripetal auditory pathways, monaurally presented sounds activate the contralateral auditory cortex to a greater extent than the ipsilateral one. In adults, non-speech sounds presented to the left ear produce more behavioral distraction (i.e., re-orienting) [40] and provoke stronger CAEP responses with shorter latencies in the contralateral hemisphere [41,42] compared to sounds presented to the right ear. The effect of this left ear advantage on behavioral reactivity and CAEP components may be explained by greater activation of the rightward-lateralized attention re-orienting network by contralateral left ear stimulation compared to the ipsilateral right ear one. It is therefore likely that putative deficits in attention re-orienting in ASD lead to greater reductions in early brain responses to left-sided (and right-hemisphere ‘addressed’) sound in the similar way as has been shown for patients with left-sided neglect [43]. Thus, a greater reduction of evoked response amplitude for temporally novel sounds (S1) coming from the left vs. right ear would support dysfunction of the right-hemispheric orienting mechanisms in ASD. Taking into account that the earliest abnormalities in children with ASD in the paired clicks paradigm were found for the P100m component [34], this component was the focus of interest in the present study.

Thus, the main goal of the present study was to explore whether neuro-functional abnormalities related to aberrant arousal/initial orienting to temporally novel sounds in young children with ASD depend on the ear that is stimulated. To achieve this goal, we investigated the P50, P100 and N1c components of CAEP in typically developing children and in those with ASD by presenting temporally novel and repetitive sounds monaurally to the left or right ear while the children watched a silent movie.

As the first step, we analyzed the amplitudes of the CAEP components. This type of analysis allowed comparison with results of our previous EEG study that applied binaural clicks [28]. As the second step, we performed a source localization analysis. This step allowed us to investigate the auditory responses separately in the left and right hemispheres. We further investigated how the CAEP indexes of abnormal arousal/initial orienting are related to the severity of autism as well as to the severity of auditory sensory modulation difficulties early in life.

## Methods

### Participants

The ASD group included 19 boys (9 boys with Asperger’s syndrome and 10 boys with autism disorder) aged 42-103 months (mean=75.3 months, SD=21.4) recruited from local psychological centers for children with developmental disabilities. The diagnosis was made by an experienced psychiatrist (VG) based on the DSM-IV-TR and ICD-10 criteria and confirmed by a clinical psychologist using the Childhood Autism Rating Scale [44]. None of the ASD children had epilepsy or any other known neurological comorbidity. All children were medication-free for at least 5 months before the examination. Their hearing was normal according to available medical records. The controls were 19 typically developing (TD) boys (age range 40-102 months, mean=76.8 months, SD=17.8) with no reported behavioral or language problems. The control children were pairwise matched to the subjects with autism by chronological age (CA). The maximal within-pair difference in CA was 11 months (mean=2.05, SD=5.1). Mental age was assessed using the Psychoeducational Profile [45] for 7 of the ASD boys (young and/or without speech) or was derived from IQ measurement with the Kaufman Assessment Battery for Children [46] for the remaining 12 boys with ASD and for all of the control participants. To specify the severity of intellectual disabilities, we calculated the developmental delay: % delay=100 – (Mental Age*100/Chronological Age). The mean developmental delay in the ASD group was 6.9% (SD=26.2, range: from -38% to +53%). According to the parent questionnaire that included eighteen questions about the child’s hand preference during everyday activities, two ASD boys and two TD children were ambidextrous and one ASD child was left-handed, while the rest were right-handed. Three of the nineteen ASD children participated in our previous MEG study [34]. Demographic information is summarized in Table 1.

**Table 1:** Demographic information.

	**ASD mean (SD), Range**	**TD mean (SD), Range**	***p*^*a*^**
	*N=19*	*N=19*	
Chronological Age (months)	75.3 (21.4), 42–103	76.8 (17.8), 40–102	*ns*
Mental Age (months)	73.4 (33.8), 23–133	89.6 (30.3), 48–144	*0.02*
Developmental delay (%)	6.9 (25.9), -37.1–53	-12.2 (15.7), -44.5–5.5	*0.005*
CARS	36 (9.4), 22–51.5	n/a	

^a^ 2-tailed T test for dependent samples.

To assess auditory sensory abnormalities, we used the questionnaire by Dahlgren and Gillberg [5] that, among others, contained six questions concerning the presence of auditory sensory modulation problems during the first two years of life. Parents assessed the severity of each problem on a 10-point scale. The same six questions were asked regarding the child’s auditory responsiveness at the time of examination (Table 2).

**Table 2:** Atypical auditory behavior at the time of examination and during the 1^st^ two years of life: mean and range of scores.

	**First two years of life**			**Time of examination**		
Question	**ASD**	**TD**	***p*^*a*^**	**ASD**	**TD**	***p*^*a*^**
*He showed strange reactions to sound*	4.9 (1-10)	1.2 (1-2)	*0.004*	2.7 (1-9)	1.2 (1-2)	*0.05*
*A hearing deficit/deafness was suspected*	3.3 (1-10)	1.06 (1-2)	*0.05*	1.6 (1-7)	1.2 (1-4)	*ns*
*He reacted strongly to sound, regardless of level*	6.2 (1-10)	3.4 (1-10)	*0.05*	5.7 (1-10)	3.6 (1-10)	*ns*
*He would often put his fingers in his ears*	2.6 (1-9)	1.2 (1-4)	*ns*	2.8 (1-9)	1.5 (1-4)	*ns*
*He sometimes reacted strongly to barely audible sounds*	3.9 (1-10)	1.2 (1-3)	*0.05*	3.4 (1-10)	1.1 (1-2)	*0.05*
*He reacted as though certain sounds were painful*	4.4 (1-10)	1.4 (1-8)	*ns*	3.3 (1-10)	1.4 (1-5)	*ns*
*Total auditory abnormality score*	25.4 (6-43)	10.0 (6-17)	*0.003*	19.4 (6-50)	10 (6-15)	*0.006*

Questions are adapted form Dahlgren & Gillberg (1989) [5].

^a^ 2-tailed Wilcoxon matched pairs test.

The auditory processing difficulties in autism are shown to decrease with age [47]. This behavioral progression, however, does not automatically indicate ‘improvement’ of the neural substrate of the auditory response but suggests that, at the behavioral level, auditory abnormalities in ASD can be better detected early in life. Therefore, we expected that more disrupted CAEP responses to clicks might be observed in those ASD children who had severe auditory modulation abnormalities during infancy and toddlerhood, even if their behavioral symptoms have diminished with age.

The study was approved by the local ethics committee of the Moscow University of Psychology and Education and was conducted following the ethical principles regarding human experimentation (Helsinki Declaration). Written informed consent was obtained from the parents of all participants.

### Procedure

During the experimental session, the child was sitting in an armchair watching silent cartoons on a 17-inch computer monitor positioned 50 cm in front of the participant. Subjects’ behavior was videotaped, and the video data were stored synchronously with the electrophysiological records. Pairs of clicks (white noise; 90 dB SPL, 4 msec in duration) were presented monaurally through wireless earphones (Sony MDR-IF140) using Presentation software (Neurobehavioral Systems Inc., Albany, California, USA). The stimuli were presented with equal probability to the right ® and left ear (L) in random order and were irrelevant to the visual presentation. The side of presentation (R or L) was always the same within a click pair. The inter-pair intervals (ISI) randomly ranged from 7 to 9 s, while the intra-pair interval was fixed at 1000 msec. The stimuli were organized into two roughly equal sessions with a 10-minute interval corresponding to the end of the first and the start of the second cartoon. In total, 170 pairs of clicks *of each type* were presented during two sessions, each lasting for approximately 30 minutes. None of the subjects displayed or reported discomfort upon presentation of the clicks.

### Data recording and analysis

Electroencephalogram (EEG) was recorded using a 32-channel SynAmps system (Neuroscan, El Paso, Texas, USA) with a linked ear reference, 0.5-100 Hz band-pass filter and 500 Hz sample rate. Four electrooculogram (EOG) electrodes were placed at the outer canti of the eyes and above and below the left eye to record horizontal and vertical eye movements. Electrode impedance was kept below 10 kΩ for all channels. The data were post hoc digitally filtered with 1 Hz high-pass and 48-52 Hz band-stop Butterworth filters. For filtering, the Matlab routine ‘ﬁltﬁlt’ (Matlab 6.5, MathWorks Inc.) was used. This routine first applies second-order Butterworth ﬁlter forward and then again backward to ensure that the phase distortions introduced by the ﬁlter are nulliﬁed.

The behavior of the participants was coded offline to identify epochs when they did not attend to the computer screen, talked or vocalized. These epochs, as well as the EEG epochs with movement artifacts or extreme signal amplitudes (±100 µV), were excluded from further analysis. EOG artifact correction was performed using a regression approach implemented by SCAN 4.2 software (Scan 4.2 System, El Paso, Texas, USA).

The EEG signal was further recalculated in an average reference montage. The data epochs, composed of 100 msec of pre-stimulus baseline and 400 msec post-stimulus EEG, were baseline corrected and averaged. There were no significant differences between ASD and TD children in the number of artifact-free EEG epochs (ASD: mean 87; SD=28; TD: 105; SD=31; p>0.05). For each subject, the average ERP waveform was calculated. Positive obligatory components (P50 and P100) of CAEP were measured at the Cz location to enable comparison with previous studies [29,48]. The components’ amplitudes were measured as an absolute maximum in the 60-90-msec window (P50) and in the 110-160-msec window (P100) after stimulus onset. As in children aged 4-8 years, the N1 wave normally shows maximal amplitude over the midtemporal regions [49], the N1c component was analyzed at T7 and T8 locations, and the N1c amplitude was defined as the absolute minimum in a 110–160-msec window after stimulus onset. A suppression percentage score for each component was calculated as [1 – (component amplitude to S2)/(component amplitude to S1)] x100, where a higher positive suppression score designates greater suppression of the component upon repetitive stimulation.

### Dipole source modeling

**Figure 1:**
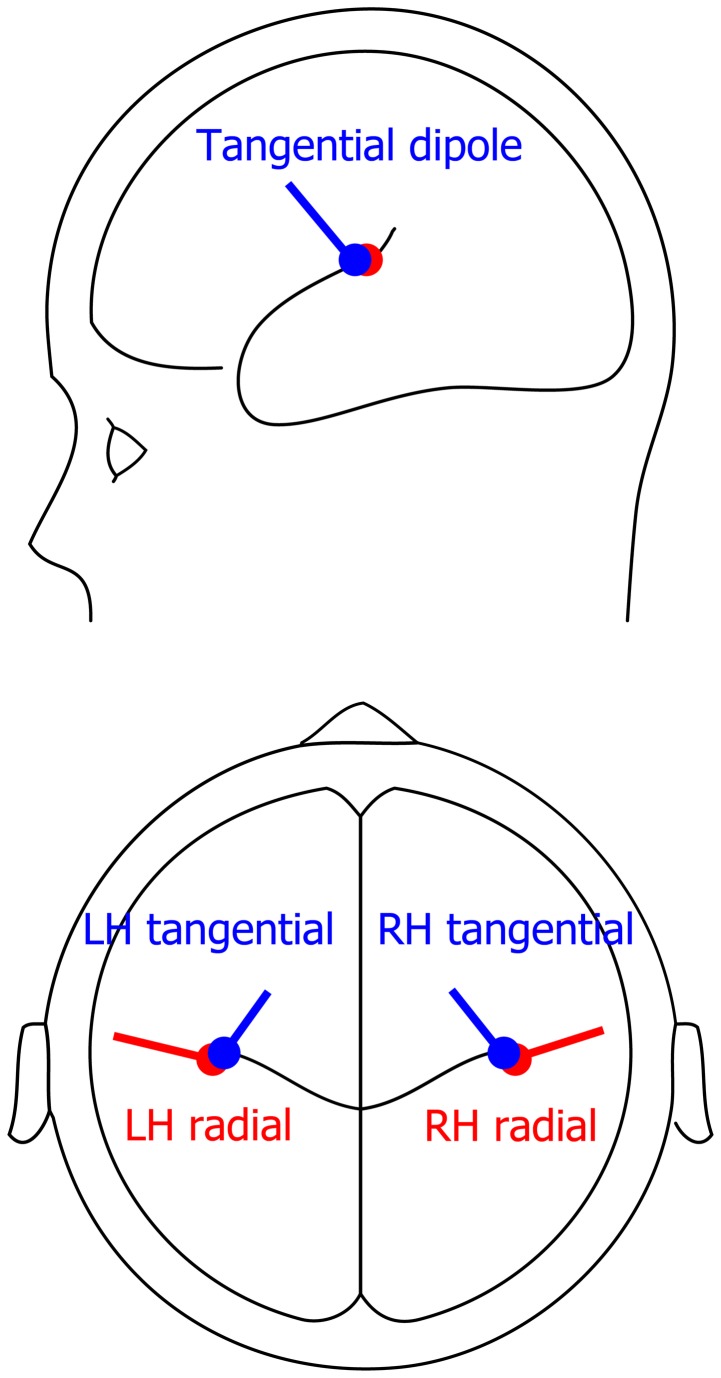
Dipole sources of grand average CAEPs in typically developing children (N=19).

The CAEPs were modeled by two symmetrical regional sources positioned in the vicinity of the left and right auditory cortices. Each regional source was represented as a linear combination of two dipole sources with a common location and orthogonal orientation. One of the two dipoles was radial according to the best-fit sphere and the other one was tangential, oriented orthogonally to the radial dipole and vertically directed. We did not include the third orthogonal sagittal dipole in the model because it explained much less of the data than the other two dipole components [50,51] and was strongly correlated with the radial and tangential dipole sources. To build the model, we used grand averaged data that have better signal-to-noise ratios (SNR) than the individual subjects’ data. To adjust the location and orientation of the radial and tangential dipoles and to insure that they represent independent cortical sources, we used the following procedure. *First*, we calculated grand average CAEP waveforms across two conditions (left and right side presentation, first click only) in TD subjects. Our use of the first click data to construct the model is justified by 1) the higher SNR of the 1^st^ click response and 2) the similar location and orientation of dipole auditory sources for short and long ISIs [52]. *Second*, we performed principal component analysis (PCA) of all electrode amplitude values in the 50-200-msec post-stimulus time window and took the first two principal components, which explained 92 percent of the variance. *Third*, we performed dipole modeling of each of the two PCA components using the Bayesian inversion approach implemented in SPM8 [53]. Initially, we fitted a symmetric pair of dipoles with strong location priors (x = ±45, y=-20z=15, MNI coordinates) and with no orientation priors. The requirement for the identical location parameters of the regional source components was relaxed during the further optimization procedure. The resulting adjusted dipole’s location and orientation were as follows: “radial” dipoles: x = ±44, y=-18z=17 and cos(X) = 0.97, cos(Y) = 0.24, cos(Z) =-0.10; “tangential” dipole: x = ±43, y=-16z=17 and cos(X) =-0.37, cos(Y) = 0.62, cos(Z) = 0.68. The goodness of fit was 87 percent for the “radial” dipoles and 93 percent for the “tangential” dipole pair (see Figure 1 for the locations and orientations of resulting dipoles).

The goodness of fit of the whole four-dipole model, assessed at the peak of the P100 component (124 msec) of the grand average waveforms for the TD group, was 91% for the first click and combined ear stimulation conditions. Thus, the model possesses good explanatory power for the TD group and allowed separation of the cortical processes corresponding to ‘tangential’ and ‘radial’ temporal sources in the two hemispheres.

The explanatory power of the TD-based four-dipole model for the grand average ASD data exceeded that for the model obtained using the combined (ASD+TD) dataset and was only slightly worse than the model derived from the ASD group itself (Table 3). Because the model obtained based on the grand average TD data possessed even better descriptive power for the grand average ASD data than the model obtained from the combined dataset (ASD+TD), we applied this model to all subjects’ data.

**Table 3:** Goodness of fit of the four-dipole models obtained using TD, ASD or combined (ASD+TD) grand average CAEPs evoked by the first click.

		**Group used to derive the model**		
		**TD**	**ASD**	**TD + ASD**
Group used for testing of the model fit	TD	91%	79%	90%
	ASD	85%	88%	79%

Note that the model derived from the grand average of the TD group explained the grand average ASD data only slightly worse than the model derived from the ASD group itself (85 vs. 88 percent explanatory power).

In the final step, the modeled dipoles were used as spatial filters (projectors) to obtain dipole activation waveforms for the individual subjects’ data [54]. When applied to an individual’s data, the grand average model picks up activity from the appropriate sources and does not minimize the overall residual dispersion, which could largely be explained by noise. For each subject and condition, the source strengths for the P50 and P100 components were determined as the maximally ‘positive’ deflection of the tangential dipole waveform within the 60-90-msec and 110-160-msec windows, respectively. The source strength for N100c was calculated as the most ‘negative’ deflection of the radial dipole waveform within the 110-160-msec latency range.

### Statistical analysis

The data were analyzed with repeated-measures ANOVAs with the factors Group (TD/ASD), Stimulation Side (L/R), Stimulation Order (S1, S2), and Hemisphere (LH/RH). The exact ANOVAs varied depending on the variables of interest (CAEP component amplitudes or dipole strength) and will be addressed in the relevant paragraphs of the Results section. To take into account differences in participants’ ages, we used a matched-subjects design. Our TD and ASD participants have been matched on chronological age, and the ordering of data has been maintained across all the analyses that included the factor Group. Note that the reported degrees of freedom reflect the number of pairs, not the number of subjects. Univariate tests for planned comparisons were applied to analyze specific differences between groups, hemispheres, or stimuli (S1 vs. S2). Dependencies of the electrophysiological measures on age, developmental delay and auditory sensory abnormality scores in the ASD group were tested using a non-parametric correlation approach.

## Results

### Differences in auditory behavior between TD and ASD children

Group differences in auditory behavior during early life and at the time of examination are summarized in Table 2. The data on auditory behavior from two ASD subjects were missing. The two groups differed on a majority of the items during the first two years of life. The total score of all of these items most reliably differentiated between the groups during early life, as well as at the time of examination. Scores exceeding maximal TD values were observed in 13 ASD children for the ‘early life period’ and in seven ASD children for the ‘current time’. The children with prominent auditory difficulties, exceeding the ASD group median either during early life or at the time of examination, did not significantly differ from the rest of the ASD sample in terms of general IQ or AQ (all t’s<1.0, all p’s>0.3).

### Components of the auditory responses to monaural clicks in TD children

**Figure 2:**
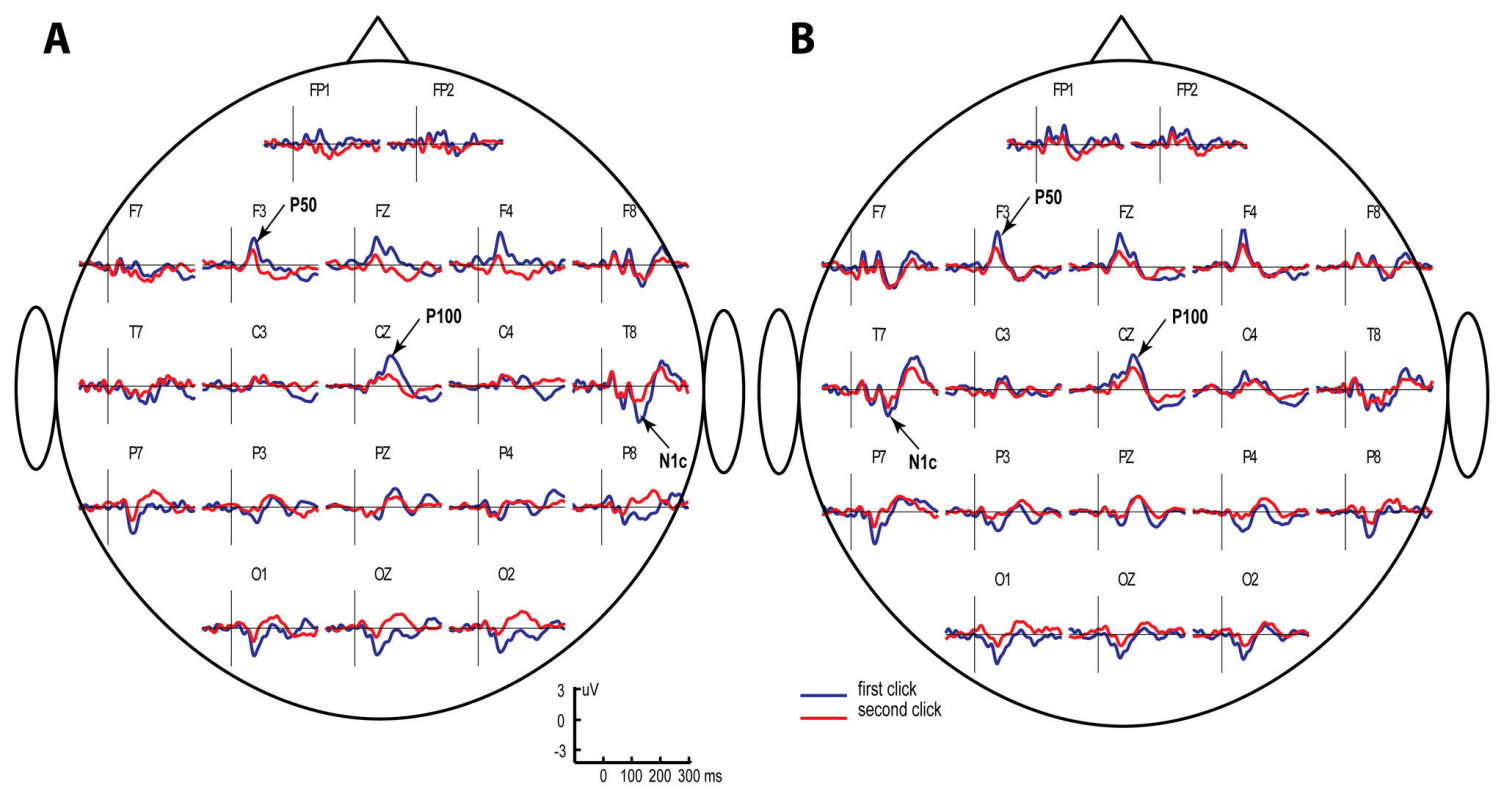
Grand average CAEP waveforms in response to left (A) and right (B) monaural clicks in typically developing children. Blue line denotes response to the first (S1) and red line to the second (S2) click in the pair. P50 wave with peak latency of approximately 70 msec is observed over frontal and central regions; P100 wave is maximal at Cz electrode location. N1c wave with peak latency of approximately 140 msec is maximal over midtemporal regions.

Figure 2 presents the grand averaged CAEPs calculated for the TD group separately for each stimulated ear and click orders in a pair.

The grand average CAEP waveforms revealed two distinct positive fronto-central peaks in the 60-160-msec time range at approximately 70 and 130 msec. The first positive peak with a maximum at the lateral frontal sites (F3 and F4) corresponds to the P50 component, which peaks within the 50-80-msec range and is known to be more frontally distributed in children than in adults [55]. The second peak had maximal amplitude along the midline and peaked at the vertex (Cz). This later positivity most likely corresponds to the P1 (also called P100) component previously described in children of this age in EEG and MEG studies [56–59]. To separate between the CAEP components observed in children at approximately 70 and 130 msec, we will address them as P50 and P100, respectively.

Apart from the fronto-central positive components, the grand average CAEPs contained a negative deflection at the temporal sites contralaterally to the stimulated ear. This temporal negativity peaks at approximately 140-160 msec after a click onset and corresponds to the N1c component of the evoked response [49], which is also called Tb [60].

### CAEP amplitudes in TD children

#### P50 and P100 waves

For the P100 amplitude, the ANOVA with factors Order (S1, S2) and Stimulation Side (left vs. right ear) revealed a highly significant Order effect (F(1,18)=14.84, p=0.001). This effect was only marginally significant for the P50 amplitude (F(1,18)=3.85, p=0.07). In both cases, the amplitudes were higher in response to the first click presented after a long period of silence. No significant effect of Side or its interaction with Order was found.

#### N1c wave

The ANOVA with factors Order, Side and Hemisphere showed a significant effect of stimulus Order (F(1,18)=5.03, p<0.04) due to a greater N1c amplitude in response to the first click. The Side of stimulus presentation had a significant effect on N1c amplitude only in combination with the factor of Hemisphere (Side*Hemisphere: F(1,18)=6.15, p<0.04). The N1c amplitude at the right hemisphere was greater for the left ear than for right ear stimulation (Left Ear vs. Right Ear: F(1,18)=10.17, p<0.006). A tendency for the greater left-hemispheric N1c in response to contralateral right ear stimulation (Right Ear vs Left Ear: F(1,18)=2.82; p=0.12) might also contribute to this interaction effect. Thus, the N1c response was mainly pronounced at the hemisphere contralateral to the stimulated ear, with the effect being more prominent for left ear stimulation.

### Dipole source modeling of auditory CAEPs in TD children

**Figure 3:**
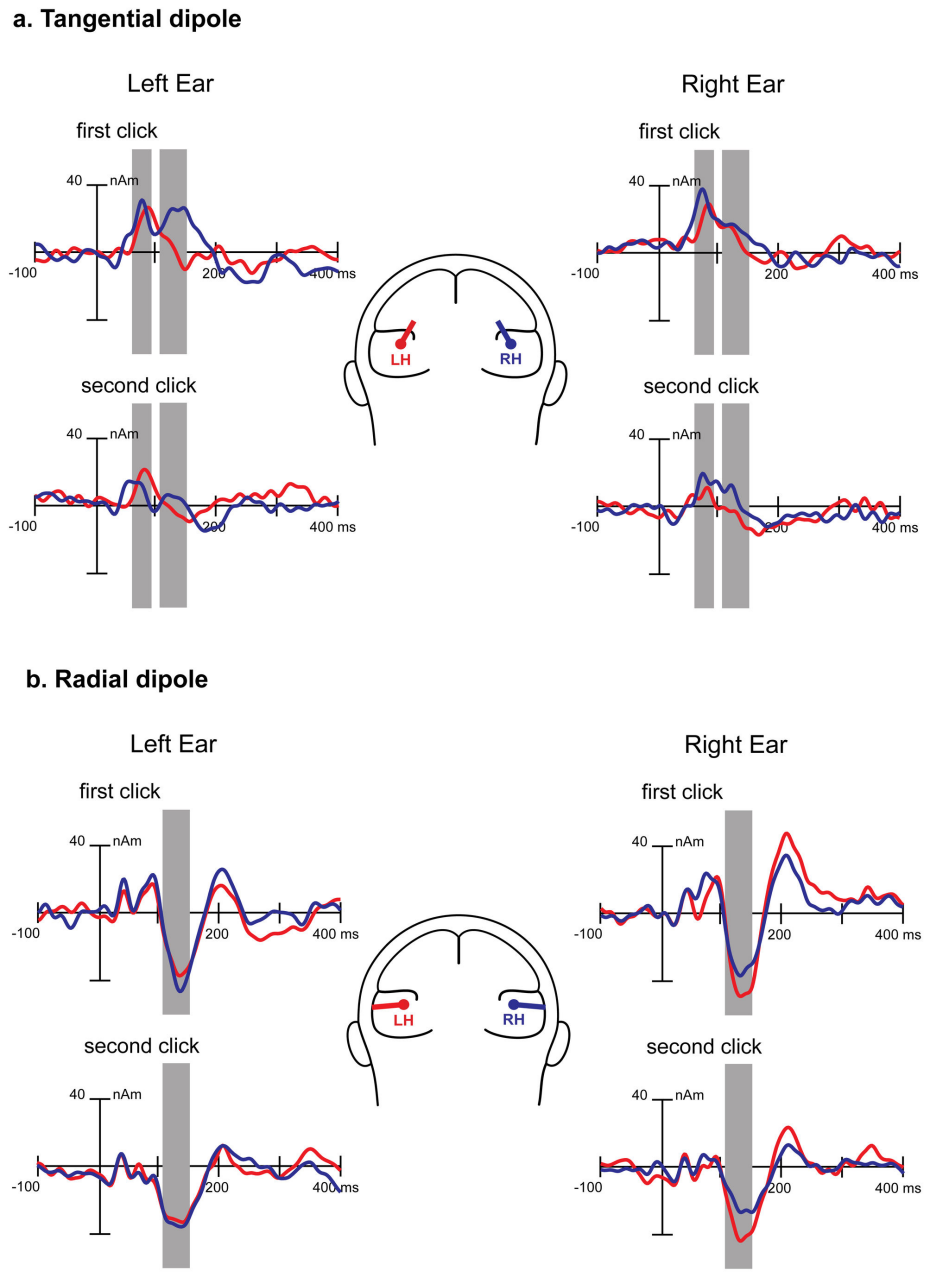
Grand average regional source waveforms obtained for the tangential (a) and radial (b) dipoles in response to the first (S1) and the second (S2) monaural clicks in typically developing children. Source activity is shown for the left (left side) and the right (right side) ear stimulation, and for the left (red) and the right (blue) hemisphere sources. The components’ time intervals taken for analysis are indicated by grey bars, referring to 60-90 msec for P50, 110-160 msec for P100 and 110-160 msec for N1c.

Figure 3 shows grand average tangential and radial dipole current amplitude time courses in the right and left hemispheres. The radial dipole source waveforms contained three distinct peaks in the time window of interest: Na, Ta and Tb (N1c) of the T-complex [51]. Below, we analyze only the most reliably identified radial component, N1c. The source waveforms of the tangential dipoles contained two positive peaks with latencies coinciding with P50 and P100. Repeated-measure ANOVAs with factors Side, Order and Hemisphere were performed for the P50, P100 and N1c dipole current amplitudes.

#### Tangential dipole sources

##### P50

The P50 tangential dipole moment showed strong main effects of stimulus Order (F(1,18)=30.90, p<0.00003). Thus, the P50 tangential dipole current amplitudes were more sensitive to the stimulus order than the traditional measure of P50 amplitude at the Cz electrode site. There was also an effect of Side (F(1,18)=5.88, p<0.03) due to higher P50 dipole amplitudes in response to the right ear stimulation than to the left ear stimulation. Concurrently, there was a tendency (F(1,18)=2.6, p=0.12) toward a greater P50 response in the right hemisphere.

##### P100

**Figure 4:**
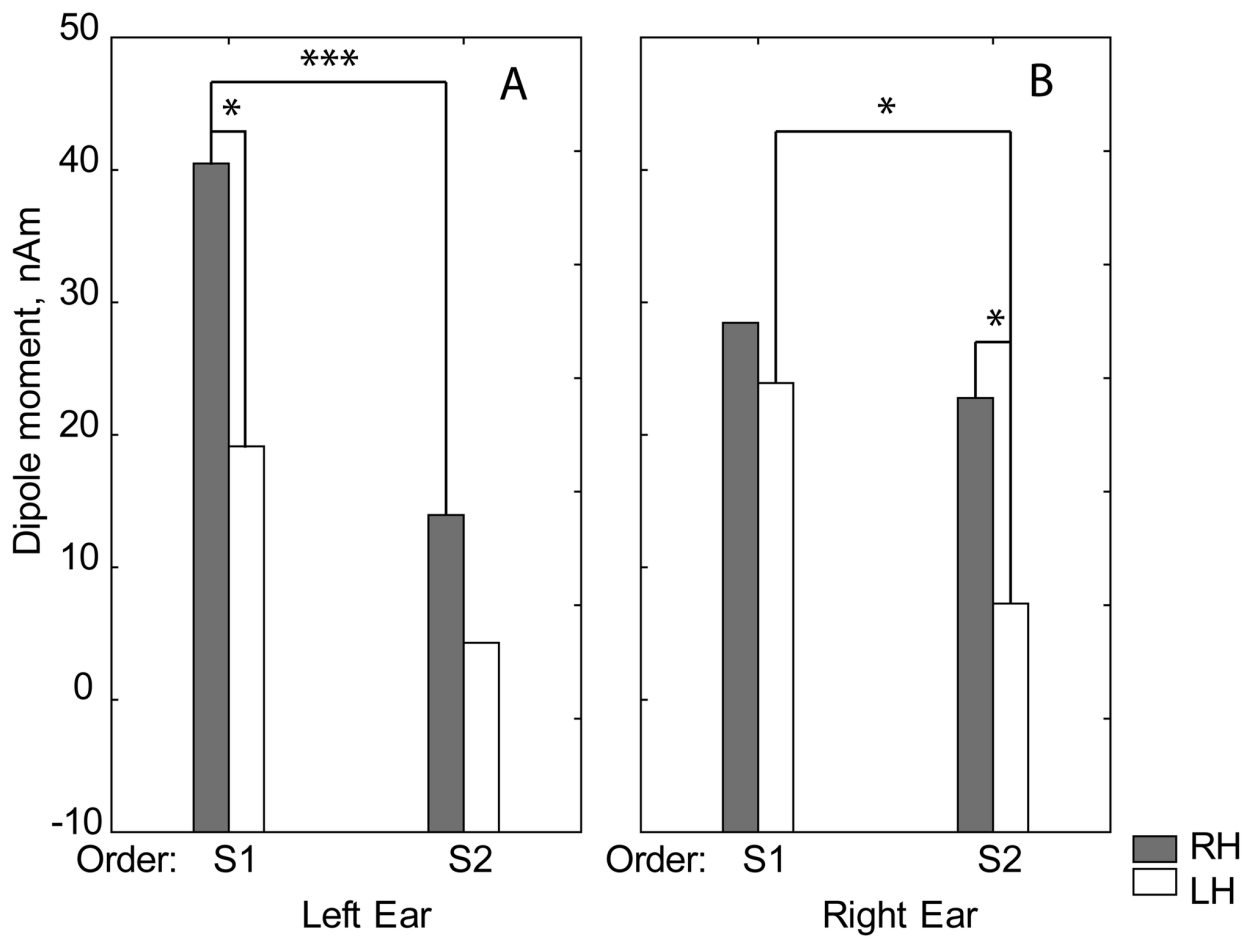
Group means of P100 source dipole moments in response to the left (A) and the right (B) monaural clicks in typically developing children. S1 vs. S2 and Left vs. Right inter-hemispheric differences for S1 and S2 stimuli; *p<0.05; ***p<0.005.

The P100 tangential dipole moment has shown a strong main effect of Order (F(1,18)=21.47, p<0.0002) due to the sharp drop in amplitude upon stimulus repetition. There was also a main effect of Hemisphere (F(1,18)=8.64, p<0.01) due to generally higher responses in the right than the left hemisphere. This rightward asymmetry tended to be more pronounced in response to the left ear than to the right ear stimulation (Hemisphere*Side F(1,18)=3.23; p<0.09), especially in response to the first click in the pair (Hemisphere*Side*Click F(1,18)=2.44; p<0.13; see Figure 4).

#### Radial dipole sources

For the N1c dipole moment, the ANOVA revealed a main effect of Order (F(1,18)=5.62, p<0.03). There was also the Hemisphere by Side interaction (F(1,18)=13.03, p=0.002) due to the stronger response in the contralateral than in the ipsilateral hemisphere. These effects are visible in Figure 3b.

To summarize, the P50, P100 and N1c components of monaural AEP in TD children were influenced by temporal novelty of the stimulation. Their amplitudes were significantly greater in response to clicks presented after a long interval of silence (S1) than in response to the S2 stimuli following S1 after a short 1000-msec delay. The tangentially oriented bi-temporal sources explaining the P50 and P100 amplitudes were relatively more affected by temporal novelty of the auditory stimulation than the radially oriented bitemporal sources ‘responsible’ for N1c. However, P50 and P100 demonstrated somewhat different behavior. The P50 amplitude in both hemispheres was more sensitive to right ear stimulation than to left ear stimulation. The P100 component, on the other hand, was characterized by general right-hemispheric asymmetry that tended to be more pronounced for a monaural click presented to the left than to the right ear.

### Comparison of CAEP to clicks in TD and ASD groups

#### CAEP amplitudes

**Figure 5:**
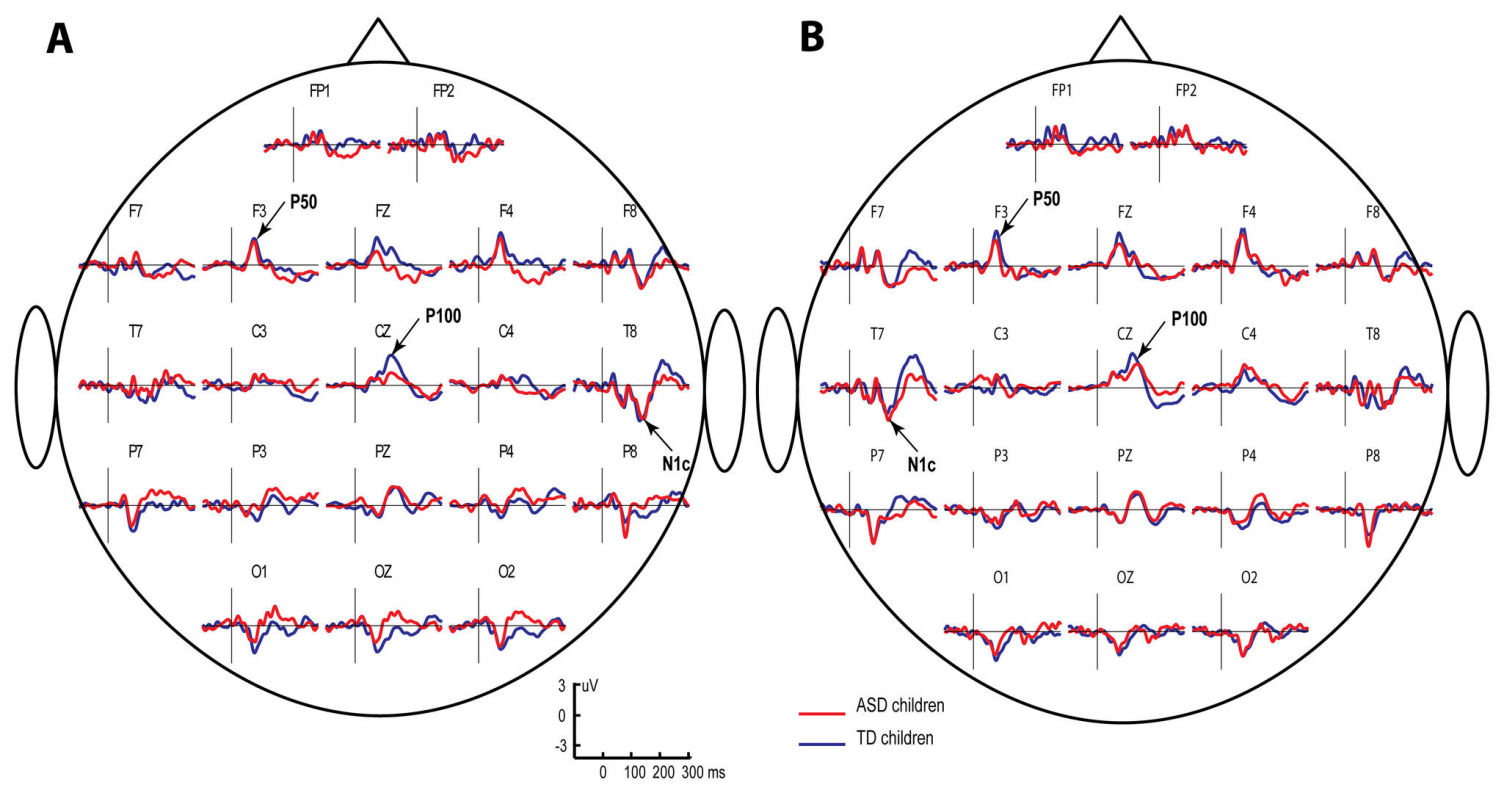
Grand average CAEP waveforms in response to the left (A) and the right (B) monaural clicks in children with ASD (red) and typically developing children (blue). Only responses to the first click in the pair (S1) are shown.

**Figure 6:**
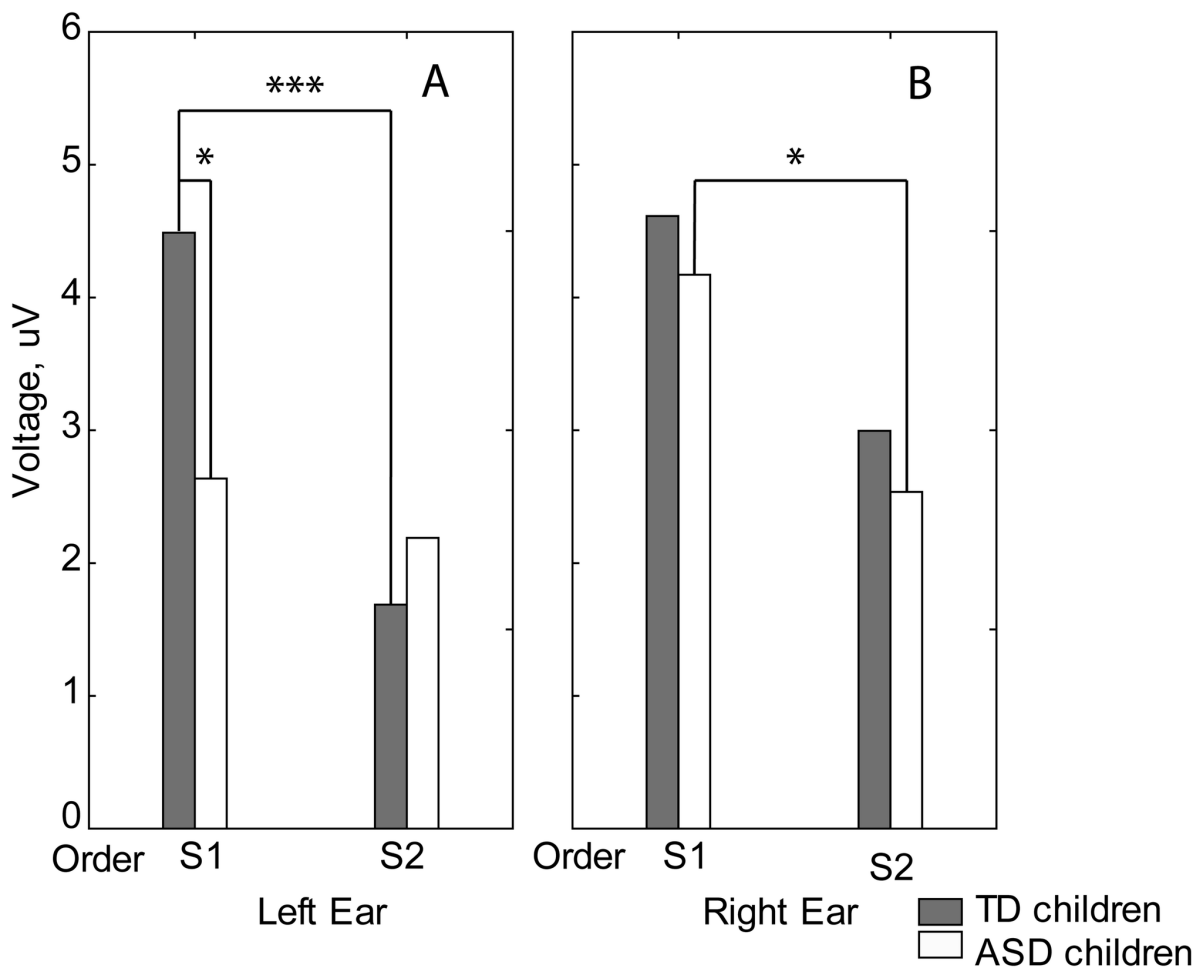
Group means of P100 amplitude at Cz in response to the left (A) and the right (B) monaural clicks in children with ASD and typically developing children. Between-group differences for S1 and S2 stimuli; S1 vs. S2 differences in ASD and control groups; *p<0.05; ***p<0.005.

Figure 5 presents grand average CAEPs in response to the first click separately for the TD and ASD groups. For the P50 or N1c components, the repeated-measures ANOVAs have revealed neither a significant main Group effect nor the interaction effects including Group factor. A significant three-way interaction of Group*Side*Order (F(1,18)=4.62, p<0.05; Figure 6) was found for P100 (Figure 6). In the TD group, the P100 repetition suppression effect was more prominent for left ear stimulation (left ear: F(1,18)=33.3; p<0.00002; right ear F(1,18)=2.21; p=0.15). In the ASD group, on the other hand, the click repetition effect was absent for the left ear stimulation (F(1,18)=0.88; p=0.36), but it was present for right ear stimulation (F(1,18)=6.23; p<0.023). The P100 amplitude in the ASD group was reduced compared to the TD group in response to the first but not to the second left-sided click (ASD vs. TD, 1st left-sided click: F(1,18)=6.23; p<0.03).

#### Dipole source modeling

**Figure 7:**
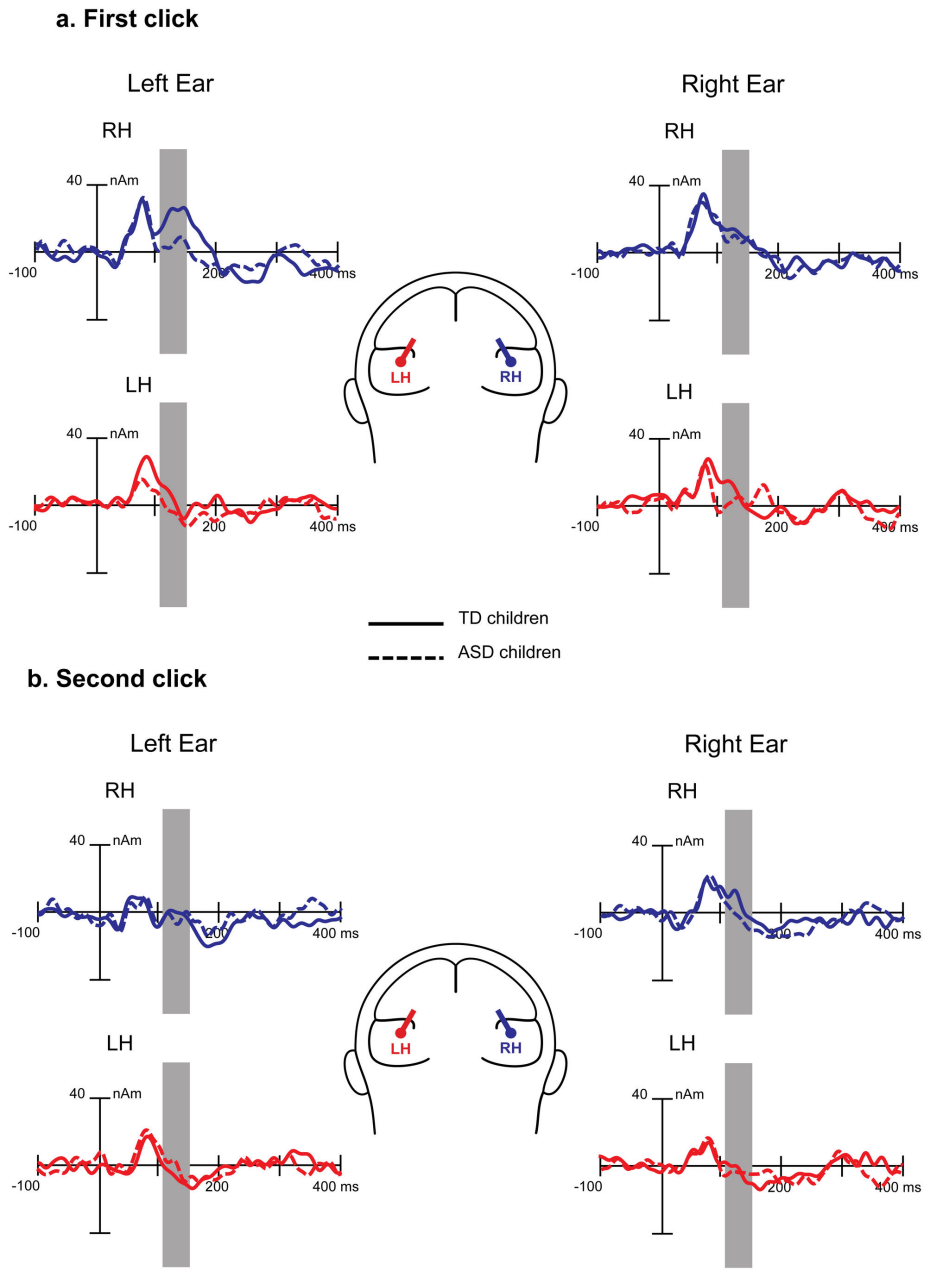
Grand average regional source waveforms obtained for the tangential dipoles located in the auditory cortex in response to the first (S1) and the second (S2) monaural clicks in ASD (dashed line) and typically developing children (solid line). Source activity is shown for left (left side) and right (right side) ear stimulation and for left (red) and right (blue) hemisphere sources. The grey bars mark the 110-160-msec window (P100) after stimulus onset.

**Figure 8:**
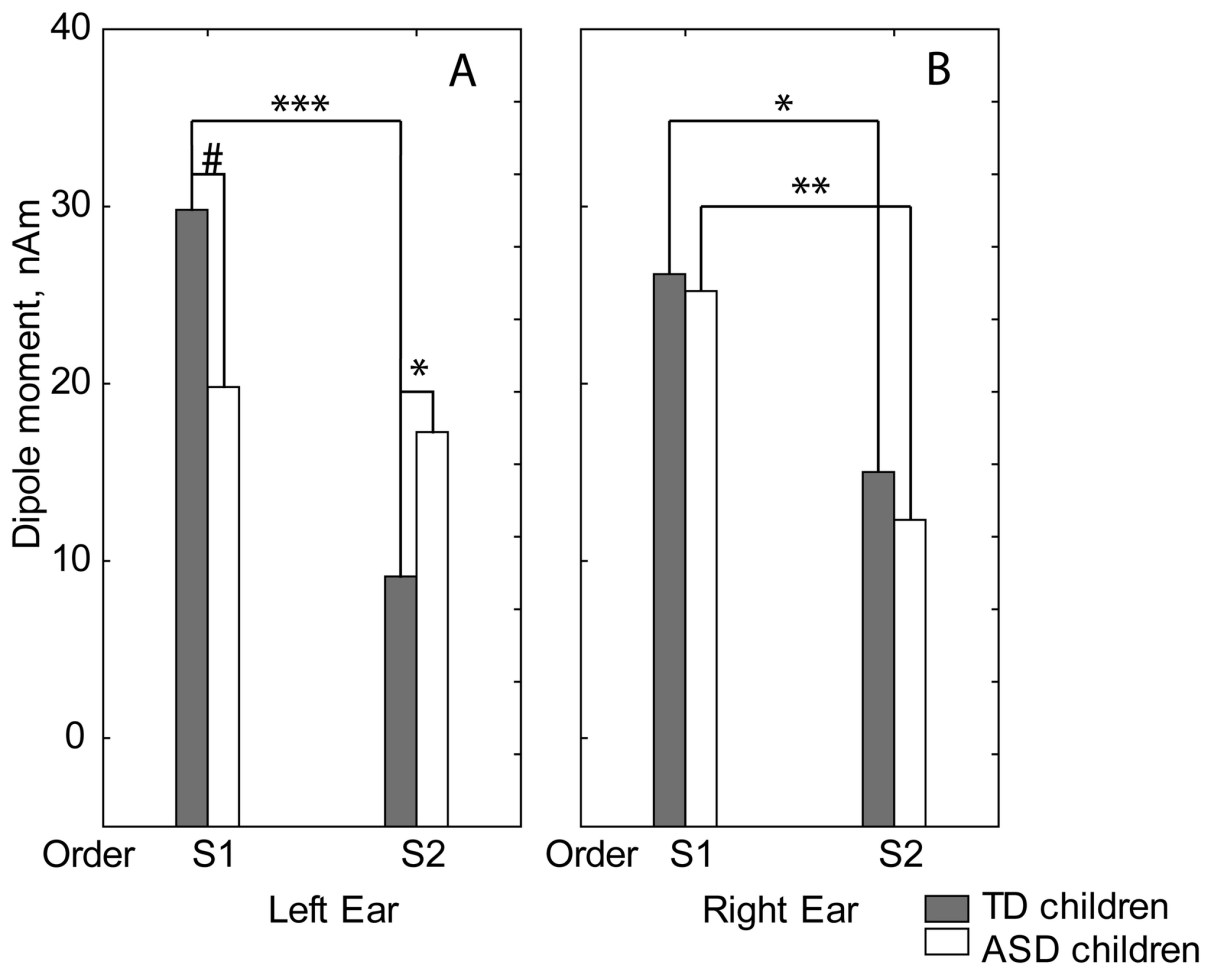
Group means of P100 source dipole moments in response to the left (A) and the right (B) monaural clicks in ASD and typically developing children. Between-group differences for S1 and S2 stimuli; *p<0.05; **p<0.01; ***p<0.005; #p=0.06.

Figure 7 presents time-varying dipole moments of the grand average waveforms calculated for tangential dipoles located in the left and right hemispheres in both groups. ANOVAs with factors Group, Hemisphere, Side and Order have been performed separately for the P50, P100 and N1c dipole current amplitudes. Similar to the CAEP waveforms, the only significant ANOVA effect including Group was found for the tangential dipole moment in the P100 time-window (110-160 msec): Group*Side*Order (F(1,18)=7.04, p<0.02, Figure 8). This interaction effect was due to the lack of P100 repetition suppression in the ASD group during left ear stimulation (first vs. second click: F(1,18)=0.58; p=0.45), in sharp contrast to extremely reliable repetition suppression during left-sided stimulation in the TD group (first vs. second click: F(1,18)=33.23; p<0.00002). Accordingly, the P100 source amplitude in response to the first left click was smaller in ASD than in TD children (F(1,18)=3.87; p=0.06), while the P100 source amplitude in response to the second left click was greater in ASD than in TD children (F(1,18)=4.69; p<0.05).

Notably, for right ear stimulation, the repetition suppression was significant in both TD (F(1,18)=5.03; p<0.04) and ASD (F(1,18)=11.18; p<0.004) groups and no between-group differences were found in response to either the first or the second clicks.

To summarize, the ASD and TD groups differed in respect to the P100 response amplitude. This difference was significant for the ‘raw’ P100 amplitude measured at Cz, as well as for the P100 dipole source strength. Compared to TD peers, children with ASD demonstrated an abnormally reduced P100 amplitude in response to the temporally novel S1 click. They also lacked P100 suppression upon stimulus repetition with a short interval (1000 msec). Most intriguingly, both the abnormal P100 amplitude reduction and the lack of repetition suppression were only found in response to the left ear stimulation, suggesting that these abnormalities are lateralized in the brains of ASD individuals.

To decrease the number of statistical tests, we limited further correlation analyses to the P100 evoked by the left monaural clicks.

### Correlations between P100 amplitude abnormalities and behavior in ASD children

**Figure 9:**
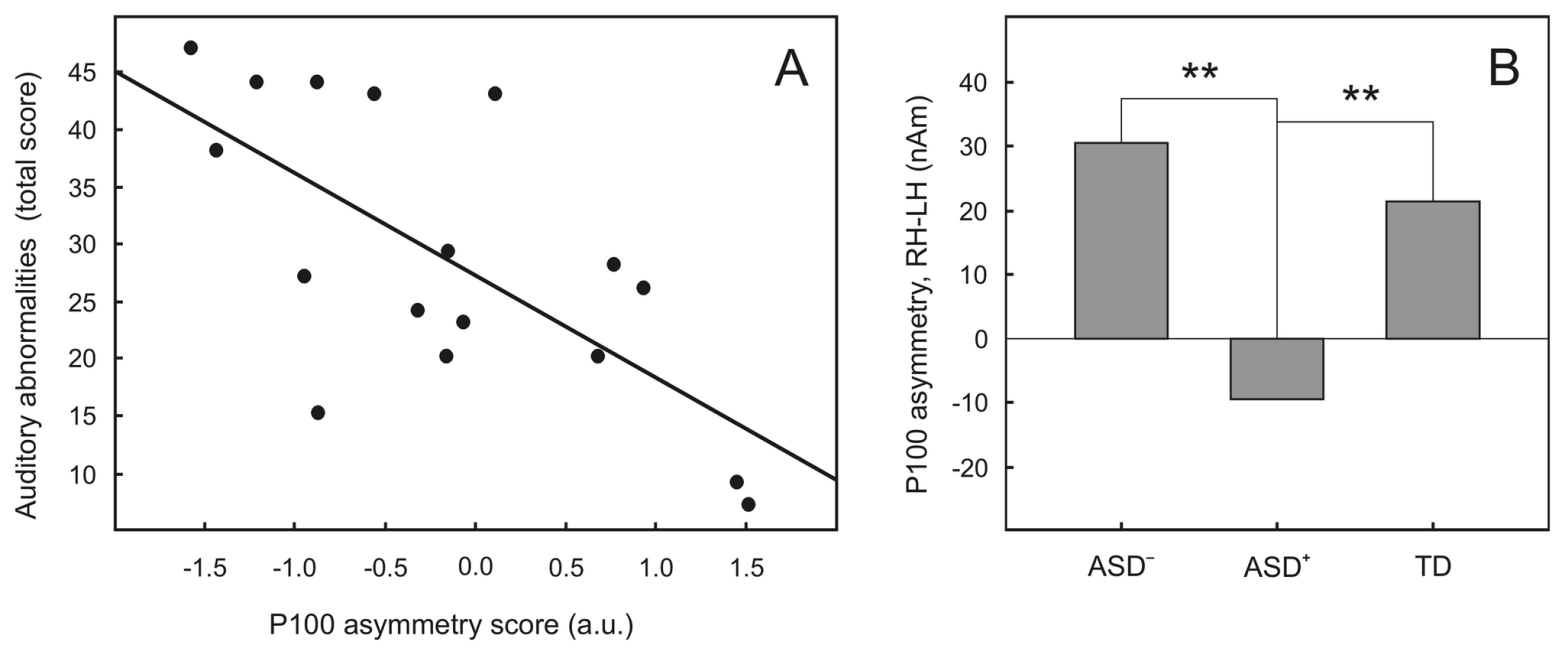
Hemispheric asymmetry of P100 source amplitude in response to the *first left* monaural click and severity of auditory sensory modulation difficulties during the first two years of life. A. Individual P100 standardized asymmetry scores (horizontal axis) vs. individual total scores of auditory abnormalities (vertical axis) in ASD participants; B. Comparison of the P100 source asymmetry scores in ASD children who experienced prominent auditory sensory modulation difficulties during the first two years of life (ASD+), in ASD children with no or milder difficulties (ASD-), and in typically developing control children; **p<0.01, 2-tailed Mann–Whitney U test.

The significant nonparametric correlations between behavioral abnormalities and P100 source strength are summarized in Table 4. Notably, the correlations between early auditory behavioral problems and P100 dipole strength were present in response to the first click only and trended in opposite directions for the hemispheres contralateral vs. ipsilateral to the stimulated left ear. The degree of auditory modulation problems in early life correlated negatively with the right hemispheric response amplitude (r=-0.55; p<0.03) but positively with the left hemispheric response amplitude (r=0.55; p<0.03). This pattern of correlations suggested that the extent of early auditory abnormalities was most reliably reflected in atypical lateralization of P100 to the first click, i.e., attenuated response in the right hemisphere in combination with enhanced response in the left hemisphere. We further computed asymmetry scores for the P100 dipole strength according to the formula (RH-LH). As expected, the asymmetry score most reliably correlated with the degree of early auditory modulation difficulties (r=-0.64; p<0.006) (Table 4 Figure 9A) and with degree of developmental delay (Table 4). No significant correlations with autism severity as assessed via CARS scores were found.

**Table 4:** Correlation^a^ between P100 dipole source strength in response to the left first monaural click and behavioral variables and age in ASD children.

	**Auditory problems during the 1^st^ two years**	**Auditory problems currently**	**Chronological age in months**	**Developmental delay (%)**	**CARS total score**
	P100 dipole strength in response to the 1^st^ left click				
Asymmetry of P100 dipole strength	-0.64**	-0.35#	—	-0.51*	—
Right hemispheric P100 dipole strength	-0.55*	**—**	0.36#	-0.42#	—
Left hemispheric P100 dipole strength	0.55*	0.51*	—	0.41#	—

^a^ Spearman rank order correlation: *p<0.05; **p<0.01; #p<0.10

To visualize the relationship between severe auditory modulation difficulties and the P100m source asymmetry, we divided ASD participants into two groups. The first group included nine subjects who scored over the median ASD group value on total auditory modulation difficulties during the first two years of life (total scores between 28 and 43). The rest of the ASD group (8 children) had no or less severe auditory sensory problems (total scores of 28 or below). Taking into account the small and unequal sample sizes, we applied the nonparametric Mann-Whitney test to compare the ‘atypical auditory sensitivity group’ with the rest of the ASD sample, as well as with the nineteen TD children. The results of this comparison are plotted in Figure 9B. The asymmetry score in the ASD sub-sample with severe auditory problems was significantly reduced both in comparison with the rest of the ASD sample (z=2.69; p<0.01) and with the TD group (z=2.33; p<0.02). While both TD children and ASD children with no or less severe auditory abnormalities had significant rightward P100 asymmetry (single sample T-tests for P100 source asymmetry in TD: T=2.81, p<0.012; in ASD: T=3.49, p<0.01), the ASD children with auditory modulation difficulties had symmetrical P100 responses (T=-0.48; p=0.64).

## Discussion

We investigated components of the CAEP to lateralized presentation of click pairs in young TD children and children with ASD. We were especially interested in the effects of temporal novelty of the auditory stimulation. Because the neural system responsible for automatic attention re-orienting to novel or perceptually salient stimuli is right-lateralized [8], we expected that the components of auditory response that reflect arousal and initial orienting to temporally novel (S1) clicks might behave differently depending on the stimulation side in the TD children. Moreover, we anticipated finding greater ASD vs. TD differences in response to the left clicks ‘addressed’ to the right hemisphere. To measure components’ amplitudes separately in the right and left hemispheres, we have undertaken dipole modeling of the auditory response sources.

The main results are as follows. In TD children, the P50, P100 and N1c components of auditory responses were strongly influenced by the temporal novelty of click stimulation and were much greater in response to the S1 click presented after a long (7-9 sec) interval than in response to the S2 click following the S1 after a 1-sec delay. The source amplitudes of ‘tangential’ P50 and P100 components and the ‘radial’ N1c component demonstrated differential lateralization. Compared with TD children, children with ASD demonstrated 1) a reduced amplitude of the P100 component under the condition of temporal novelty (S1 click) and 2) an attenuated P100 repetition suppression effect. These abnormalities were evident for clicks presented to the left but not to the right ear.

Taking into account the scarceness of literature describing characteristics of monaural CAEP responses to left vs. right-sided clicks in children, we will first discuss the morphology of these responses in the TD group and will then address between-group differences.

### Morphology of CAEP evoked by monaural clicks in typically developing children

#### Components of child CAEP to temporally novel clicks in TD children

The developmental literature has consistently shown that CAEP components are not fully mature until adolescence, and the morphology of the CAEP response in children is strikingly different from that in adults [51,57,61–63]. Moreover, the stimulus characteristics and rate of stimulus presentation affect the CAEP components in children to an even greater extent than they do in adults. Some of the child CAEP components may be substantially attenuated or even disappear upon condition of relatively rapid succession of auditory stimuli (less than 2-sec intervals between stimuli), whereas long inter-stimulus intervals favor discrimination of the CAEP component [62,64]. Despite this fact, the majority of developmental studies employed short ISIs to characterize the obligatory waves provoked by auditory stimuli [50,57,58,65]. From this perspective, long (7-9 sec) intervals of silence before the first click in our study might favor discrimination of CAEP components.

In our TD subjects, responses to clicks were characterized by three main components in the 50-200-msec range. The first component (P50) was a positive peak with a latency of approximately 70 msec and a maximum amplitude at the fronto-central electrode sites. This component was followed by the second positive wave of greater amplitude (P100) at approximately 130 msec, which reached its maximum at the vertex (Figure 2). These two positive components belong to the ‘P1-family’ and, in good accordance with the previous dipole modeling results [50,51], are satisfactorily modeled by two tangential dipoles symmetrically positioned in the temporal lobes of the left and right hemispheres.

The third negative component with a peak latency of 140 msec was clearly visible at symmetrical midtemporal recording sites. This component is commonly labeled Tb [60] or N1c [49]. Two radial temporal dipoles provided the best fit for N1c, which corresponds well to the literature [50,51,66].

Previous developmental CAEP studies typically reported only one peak in the ‘P1’ time range (50-130 msec) in children. Therefore, our finding of two positive ‘P1’ components deserves separate discussion.

The majority of developmental EEG and MEG studies that presented auditory stimuli with short (less than 2 sec) inter-stimuli intervals (ISI) found that the broad wave within 50-130 msec is the only Cz-positive obligatory component in children and considered it as a developmental homolog of the adult P50 [50,58,59,62,67]. At the same time, a few EEG and MEG studies that, similar to the present study, employed a paired click paradigm with long inter-pair intervals and sharp onset stimuli consistently reported a positive wave with much shorter, nearly ‘adult’ latencies of 50-80 msec in children [28,55]. Moreover, intracranial recording confirmed that the P50 wave at approximately 50 msec could be observed in the auditory cortex, even in 3-year-old children [68]. This discrepancy in findings suggests that the adult-like P50 is present even in young children, but it may be more difficult to detect in children than in adults because of its poor signal to noise ratio and/or its partial overlap with the later P100 positivity. Notably, our recent MEG study [34] has shown that the MEG analog of P50 in children might be more easily detected in response to monaural than binaural clicks due to its smaller overlap with the later P100m positivity during monaural than binaural stimulation. Monaural stimulation in the present EEG study may therefore favor separation of the P50 from the later P100 positivity.

Our findings clearly show that P100 is separate from the P50 CAEP component in children aged 4-8 years. Moreover, the findings suggest that these two components may be related to different neural processes in the auditory cortex.

Although in adult CAEP studies the positive wave within 50-80 msec is commonly referred to as the P50 component, some studies also reported two components ‘Pb1’ and ‘Pb2’ in this time range in MEG [69] and intracranial EEG records [70,71]. It is conceivable, therefore, that the P50 and P100 waves in preschoolers may represent the developmental analog of adults’ Pb1 and Pb2 [69] CAEP components.

The presence of the two components in the P1 time range in children raises a possibility that developmental changes in ‘P1’ latency and amplitude may at least partly reflect an alteration in the relative prominences of P50 and P100 with age. This assumption can help us to better understand the developmental dynamics of the ‘P1’ positivity described by Ponton et al. [51]. Ponton et al. [51] have argued that maturational changes in amplitude and latency of the ‘tangential P1 component’ can be explained by the gradual emergence of an obligatory ‘adult’ N1b component rather than by maturation of the P1 generators. This conclusion was based on studies in animals that showed that the animal analog of P1 originates from early maturing infragranular layers of the auditory cortex, while the following negativity (N1b) results from activation of more superficial supragranular layers starting their maturational spurt in humans only after 5 years of age [72]. Due to the temporal overlap and similar tangential orientation of the N1b and the preceding positivity, the magnitude and latency changes of the maturing N1b peak are superimposed on the mature magnitude and latency properties of the P1 [51]. As a result, from 8–10 years old and onward, the later part of the early maturing P1 starts to be partially cancelled out from the surface EEG, despite the fact that the neural processes underlying this component are fully functional. Our present results suggest that this ‘later part’ is dominated by the P100 component.

Due to partial P100 cancellation, diminishing latency differences between P50 and P100 toward adulthood, as well as because of the filtration parameters applied, the two consecutive waves may produce a single peak in adult CAEP responses (labeled by different authors as P1, Pb or P50), as has already been assumed by some authors [70,73]. From this perspective, the typical properties of the P100 wave, as well as its neurofunctional abnormalities in populations with developmental disorders, could be adequately studied only in children. However, as the magnitude and latency of the N1b at this age are blurred by more mature P100, it is difficult to derive any reliable conclusions about the effects of experimental manipulation of N1b in young children.

#### Repetition suppression of CAEP components in TD children

As the ISI shortened from 7–9 sec to 1 s, the amplitudes of all investigated obligatory CAEP components (P50, P100, and N1c) and the respective dipoles source strengths decreased, regardless of the stimulated ear (Figure 2 Figure 3). The presence of this repetition suppression effect is generally consistent with the previous developmental literature [48,62,64].

In our developmental study, the positive components of P50 and P100 seem to show even greater repetition suppression effects than the N1c (a suppression percentage score for the left clicks: 52.4% dipole strength reduction for P50, p<0.0001; 82.3% for P100, p<0.0001; 30.4% for N1c, p<0.03). The neural processes underlying the generation of positive CAEP components may shed some light on this finding. The obligatory P1 in humans is most likely generated at the lateral belt and parabelt regions of the superior temporal gyrus (STG), i.e., outside the primary auditory cortex [74], and in the immature cortex it reflects primarily bottom-up activation of these areas [75]. Unlike the primary auditory cortex, which receives the main thalamic input from specific lemniscal pathways, the auditory belt and parabelt areas generating P1 are more sensitive to noise stimuli than to pure tones and are predominantly fed by extra-lemniscal auditory input from non-specific thalamic nuclei, such as the medial pulvinar, nucleus limitans and suprageniculate nuclei [76], i.e., from thalamic structures that are thought to be involved in arousal and attention regulation [77,78]. It is conceivable, therefore, that the heightened sensitivity of both the P50 and P100 components of child CAEP to temporally novel (S1) stimulation in our study was due to their dependence on input from non-specific thalamic nuclei involved in arousal and attention regulation.

The contribution of phasic arousal to P50 and P100 is generally consistent with the well-documented contribution of cholinergic ascending input to the auditory cortex in P50 (P1) generation [35]. This role is also supported by findings of the amplifying effect of nicotine – a drug that stimulates the cholinergic branch of the ascending activation system – on the P1 response to S1 in the paired click paradigm [26]. The nicotine effect in the Rudnick et al. study could be related to either of the P1/Pb sub-components (Pb1 or Pb2), given that P1 (Pb) in this and other ‘paired-click’ studies was defined as the largest positive deflection with a maximum within the 40-75-msec window, which most likely covers both Pb1 and Pb2 waves in adulthood [69]. If two approximately equal amplitude peaks were present in the data around the P50 latency, researchers may pick up either the earlier or the later for the P1 analyses. It is interesting that chronic nicotine exposure, which provokes robust nicotinic cholinergic receptor up-regulation and to heighten cholinergic arousal [79], seems to primarily augment in adults the second positive wave – Pb2 (75 msec), as it shown by Wan and colleagues in their Figure 1 [80].

Given the findings summarized above, we assume that the augmented P50 and P100 responses to S1 in children may reflect consecutive waves of cholinergic modulation of thalamocortical and intracortical transmission in auditory pathways. Still, the P50 and P100 properties may reflect different aspects of cholinergic modulation, which is known to operate through multiple anatomical routes [81] and/or through nicotinic or muscarinic regulation of auditory responses [82].

#### Hemispheric lateralization of CAEP components in TD children

The pattern of hemispheric lateralization in response to monaural clicks differed for P50, P100 and N1c components. For both left and right monaural clicks, stronger N1c responses were observed in the hemisphere contralateral to the stimulation.

The contralateral effect, i.e., a relatively greater amplitude of monaural N1b CAEP component source in the hemisphere contralateral vs. ipsilateral to the stimulated ear, is commonly described in the literature on adults [18,83,84]. Moreover, our N1c findings are generally compatible with the results of other developmental studies that showed greater N1c amplitude in the contralateral hemisphere while stimulating only either the left ear [51,57,60] or right ear [85,86]. Our study contributes to the scarce developmental findings [87] that allow direct comparison of the effects of left and right ear stimulation on child CAEP.

Unlike N1c, the positive components showed rightward hemispheric asymmetry, regardless of the stimulated ear. This rightward predominance was especially strong for the P100 component. Rightward lateralization of the obligatory P50/P50m component sources has been previously reported in some sensory gating studies that applied binaural clicks in adults [88–90] and children [34]. The rightward hemispheric asymmetry has also been reported for P100m in children [34]. Thus, the rightward lateralization of the P100 in response to temporally novel clicks appears to be a rather stable developmental phenomenon.

It has been shown that morphological structure of auditory cortex may result in cancellation of evoked potentials and magnetic fields generated by ‘tangential’ sources and that this cancelation is greater in the left vs. right hemisphere [91]. Therefore, the rightward predominance of P50 and P100 found in TD children in the present study may, at least partly, be explained by morphological differences in the left and right auditory cortices. The functional differences, however, are also likely to play a role. Thus, it has been suggested that sharp ramps of the sound may contribute to the rightward lateralization [92]. The other property of click stimulation that could contribute to the rightward P100 lateralization is its tendency to evoke phasic cortical arousal and potentially - re-orienting of spatial attention. Supramodal specialization of the right cortical hemisphere for arousal and attention orienting has been consistently shown in neuropsychological and fMRI studies [9,93]. In case of auditory stimulation this specialization seems to exist already at the level the secondary auditory cortex. Thus, the recent fMRI study has shown that during auditory spatial orienting task the planum temporale was activated greater in the right than in the left hemisphere irrespective of the stimulated ear [94]. The role of arousal processes, predominantly subserved by nonspecific thalamic input to the *right* hemisphere, may be especially important for rightward lateralization of the CAEP components in response to the inherently salient, temporally novel, but unattended S1 click. Indeed, in the case of left ear stimulation in the TD children, rightward hemispheric asymmetry of the P100 component sources tended to be greater for the S1 than for the S2 click (Hemisphere*Side*Click F(1,18)=2.44; p<0.13; Figure 4).

Contribution of stimulus characteristics (temporal novelty, sharp ramps) into the rightward lateralization of P100m to the S1 click is further supported by the fact that the opposite, leftward lateralization of this component has been found in children in case of relatively fast (stimulus–onset asynchrony of 3 seconds) presentation of noises and violin tones [56]. The leftward lateralization of P1m (at 65 ms) also has been found in adults in response to noises presented with short intervals (<= 2 sec) and characterized by soft ramps (15 ms) [95]. The P1m (at 93 ms) to speech sounds in children is also leftward-lateralized [96].

### Attenuated rightward-lateralized P100 response in autism

#### P50, P100m and preattentive arousal in ASD

The present EEG study provides evidence of an altered P100 cortical response to monaural clicks in young children with ASD. The main findings are the P100 reduction in response to the temporally novel (S1) clicks presented to the left ear and attenuated P100 repetition suppression in response to left ear stimulation in ASD (Figure 6). The latter effect potentially depends on a decreased response to S1, increased response to S2, or both. In our ASD subjects, both types of analysis (CAEP amplitudes and dipole modeling) convergently demonstrated atypically *decreased* P100 responses to S1 (Figure 6 Figure 8), while dipole modeling alone pointed to an abnormally *increased* response to S2 during left ear stimulation (Figure 8). Given the possible pitfalls of dipole source modeling, especially in the case of the relatively poor signal-to-noise ratio of the S2 response, the reliable conclusion drawn from our data is that autism spectrum disorder is characterized by a decreased amplitude of the S1 response, while the S2 amplitude effect has to be confirmed by future studies.

In contrast to the clearly abnormal behavior of the P100 wave, the earlier positive P50 component was not affected in ASD children and displayed typical amplitudes in response to the S1 click (Figure 5 Figure 7), as well as a typical repetition suppression effect (Figure 7). The present data fully agree with the previous studies reporting normal P50 gating in children with autism [29,30], unless they had prominent mental retardation [31].

On the other hand, Buchwald et al. [97] have found that adults with autism and normal IQ displayed reduced positivity in the latency range of 50-65 msec at a relatively slow click presentation rate of 0.5/sec. The discrepant P50 findings in studies on adults and children might be explained by developmental progression of the neural processes underlying ASD, although the role of between-studies differences in experimental paradigms and filtration techniques cannot be excluded.

Whatever the reasons for the abovementioned discrepancy, our present findings, as well as Buchwald et al. [97] results, point to alterations in the obligatory positive CAEP components’ responses to clicks in ASD. In view of the strong physiological arguments for the link between these components and the cholinergic arousal provoked by auditory clicks [98], our current finding of P100 response abnormalities suggests that dysfunction within the cholinergic non-specific system is an important contributing factor to preattentive auditory orienting deficits in autism. At the same time, the absence of similar abnormalities for P50 in our pediatric sample strongly suggests that the P50 and P100 components reflect different functional aspects of a larger cholinergic modulatory system.

#### Implication of preattentive arousal deficits in behavioral orienting abnormalities in autism

Detection of new events occurring outside the focus of attention is fundamental to adaptive functioning and is most critical when attention is focused elsewhere. The unattended sensory events may demand further analysis according to their task relevance and may appear important for survival. From this perspective, our findings the autistic brain becomes, to a certain extent, impenetrable to temporally novel events in the auditory sensory modality when involved in the processing of visual stimuli. Such a view, if correct, would be concordant with attention switching difficulties to unattended sound as reported in clinical settings. This view is also consistent with the results of the behavioral studies by Courchesne et al. [99], who demonstrated that autism disorder is characterized by slow orientation to stimuli across sensory modalities and slow shifting of attention [99–101]. More recent behavioral literature concentrating mainly on the visual modality [102] confirmed the main conclusion of the previous studies about the slowing of switching (disengagement) attention in autism, although it points to a strong dependence of the behavioral findings on the methodological details of the attentional paradigm.

Cognitive evaluation of deviant or unexpected events is reflected in the P3a component of event-related responses that is usually measured in ‘novelty oddball’ or ‘passive oddball’ paradigms [14]. Strikingly discrepant P3a results were obtained in children with ASD using non-speech auditory stimuli. The P3a-like response to highly deviant unique stimuli (novels) embedded in a sequence of repetitive (standard and deviant) sounds was strongly reduced in children with ASD [15,16]. On the other hand, the studies that employed other types of the oddball paradigm (e.g., passive ‘oddball’, non-unique ‘novels’, etc.) reported unchanged [103,104] or even increased [105–107] P3a amplitudes in children with ASD. As Whitehouse and Bishop [108] noted, such inconsistencies might arise from a strong dependence of the P3a amplitude on a number of experimental factors varying between different studies, e.g., the nature of the repeating sounds that precede the novel stimulus. Indeed, although P3a is thought to be associated with involuntary switching of attention toward stimulus changes occurring outside the attention focus [109], this long-latency and widely distributed response mainly reflects a rather late evaluative stage of information processing, which is modulated by the familiarity of the stimulus and the context within which the novel event is embedded [14]. Any changes in P3a may therefore be a consequence of the disorganized higher-level psychological processes characterizing ASD. In contrast, the P100 reduction observed in the present study suggests that abnormal reactions to novel unattended events in children with ASD stem from disturbances of the early preattentive processing stage, which mostly depends on innately arousing features of unattended sound.

#### Lateralized abnormalities in ASD: evidence in favor of hemi-spatial neglect

Why did we find impairment in modulation of the auditory response to S1 only when the sound was presented to the left ear, but not to the right ear? According to the neurophysiological model of arousal [110,111], the arousal pathways mostly target the right hemisphere, which is fed mainly by projection from the contralateral left ear. Indeed, in typically developing children, the right-lateralized P100 response to the first click tended to be more pronounced for the left compared to the right ear stimulation (Figure 4). If the view of malfunctioning RAS thalamo-cortical cholinergic pathways in autism is correct, then it is not surprising that the auditory P100 response, which is triggered by left-side auditory stimuli and is highest in TD children in response to the arousing S1 click, would show maximal abnormalities. Following this line of reasoning, it is tempting to speculate that diminished P100 in response to left-sided, but not right-sided, unattended sound may indicate lateralized imbalance in early orienting mechanisms in ASD in a similar way as has been proposed for patients with left-sided neglect [111].

The deficiency of involuntary orientation toward left-sided peripheral stimuli in neglect patients is thought to be caused by damage to the cortical right-hemispheric attention disengagement system that extricates attentional focus from the previously attended location to the unexpected stimulus in the left hemi-space [8]. Another suggested origin of left-sided neglect is a dysfunctional arousal system, which leads to a partial failure of right-lateralized cortico-petal projections of the subcortical arousal nuclei to fully engage the cortical attentional system in a data-driven manner [111,112]. It is interesting that left-sided unilateral neglect in patients with right brain damage is causally linked to both decreased arousal and its electrophysiological index, diminished auditory P1 response to binaural S1 clicks in the S1-S2 paradigm [113]. Two available studies of monaural auditory evoked potentials in neglect patients [43,114], although focused exclusively on mismatched negativity analysis, still presented ERP waveforms [43,114] showing reduced P1 in response to left-sided compared to right-sided auditory stimulation. This asymmetrical auditory P1 attenuation in neglect patients closely resembles our finding in young children with ASD and is in line with our speculation on similar neural deficiencies in the two clinical populations.

Unlike neglect patients, ASD individuals do not have grave structural right-hemispheric abnormalities, and their disengagement deficit is more likely to be explained by functional dysregulation of the attention re-orienting network. It has been previously suggested that a failure of the nicotinic cholinergic neurotransmitter system may be an important factor contributing to attention abnormalities seen in ASD [36]. This system is critically involved in attention disengagement to peripheral targets [38,39] and has a ‘left hemi-space bias’. In both monkeys and humans, nicotine mainly speeds re-orienting to peripheral stimuli that appear in the left hemispace and are processed primarily by the right hemisphere [39]. Because a deficit in nicotinic receptors is well documented in ASD individuals [36,115–117], this problem may contribute to both left hemi-space lateralization of the P100 abnormalities in children with ASD in our study and to attention re-orienting/disengagement problems observed in ASD in a number of previous behavioral studies [118].

In brain-damaged patients, injury of the right hemisphere and dysfunctional upward and downward projections between the non-specific subcortical nuclei and cortical regions involved in attention regulation are proposed to be complementary causes of unilateral neglect [111]. In much the same way, disturbances of the subcortical-cortical loops within the neural network subserving exogenous orienting in children with ASD may result in dysfunctional arousal processes indexed by unilateral reduction of auditory P100. If ASD and hemispatial neglect syndrome do have some commonalities in their neural substrate, one may expect sub-clinical symptoms of left-sided neglect to be observed in children with autism.

Bryson et al. [119] have previously hypothesized that children with autism suffer from a subtle form of developmental unilateral visual-spatial neglect. Similarly, Casey et al. [120] have found that autism savants had particular difficulty with disengaging and shifting attention to the left hemi-space. Although disengagement deficit in ASD individuals has been subsequently replicated in many studies across life spans [102], its dependence on the visual hemi-field either has not been studied [121] or was shown to be bilateral [122]. It is likely, however, that specific attributes of the experimental task applied to uncover sub-clinical symptoms of behavioral neglect in ASD may be essential to find the lateralized deficit. For example, in patients with a sub-clinical form of left unilateral neglect, the left-sided extinction was evident only during high attention load at a fixation point [123]. The lateralized difficulties with attention disengagement in young children with ASD may also depend on the extent to which attention has been engaged by the previous spatial location. In line with this assumption, the recent behavioral study of children with autism aged 3-5 years did reveal atypical right-sided bias in their performance of two difficult spatial working memory tasks, which both required attention to be switched either to the right or to the left from the previous strongly attended location [124].

Kawakubo et al. [125] provided the first electrophysiological evidence for dysfunction of the attentional disengagement system in autism. They found abnormal pre-saccadic potential in adults with autism during performance of a task requiring gaze shifts to peripheral targets. The authors used two experimental conditions. During the first one the central fixation stimulus disappeared before presentation of the peripheral target stimulus (‘gap’ condition). Under the second condition the central fixation stimulus was left on the screen during peripheral target presentation (‘overlap’ condition). In order to execute saccade to the peripheral target under the ‘overlap’ condition, participants had to disengage attention from the central stimulus, while such disengagement was not necessary in the ‘gap’ condition. The atypically high pre-saccadic positivity in subjects with autism was found only under the ‘overlap’ condition and has been assumed by the authors to reflect the allocation of extra effort for attentional disengagement. Interestingly, the authors reported a significant ANOVA Side vs. Condition interaction effect, which is illustrated in their Figure 3 [125]. Their results show that enhanced pre-saccadic positivity in subjects with autism was evident only under the condition of *left* peripheral visual stimulation. This finding means that subjects with autism allocated more resources to divert their gaze to the left peripheral stimulation compared to the right one. Such asymmetry is in line with our present finding of lateralized auditory P100 abnormalities and also suggests a parallel between individuals with autism and left spatial neglect patients.

To summarize, our finding of a left ear bias in P100 response deficiency in ASD points to a lateralized imbalance in early orienting mechanisms and is generally consistent with previous behavioral and electrophysiological results. This finding indirectly supports the assumption of Bryson et al. [119] about subtle forms of left unilateral neglect in children with autism.

#### Preattentive arousal deficit and auditory modulation difficulties in ASD

We suggest that the impairment of the preattentive arousal processes indexed by the P100 reduction may underlie several well-known auditory-related abnormalities in autism, including hyper- and hyposensitivity to sounds, as well as abnormal orienting to auditory events.

Many of the ASD children in our sample had auditory modulation difficulties in early life and, to a lesser extent, at the time of investigation (Table 2). These behavioral findings agree with previous behavioral studies [6,47,126–128]. We expected that a child’s problems with auditory processing in infancy, being more evident for caregivers, might be related to P100 attenuation in childhood. In line with our prediction, the severity of early auditory abnormalities was inversely related to the P100 source strength attenuation in the right hemisphere under the condition of left ear stimulation (Table 4). This finding corresponds well with the recently reported association between heightened auditory sensitivity and reduced volumetric gray matter growth in the right hemisphere in children with ASD [129]. The unexpected finding was the opposite positive correlation of auditory abnormalities with P100 strength in the left hemisphere. As a result of these opposite correlations, the behavioral auditory modulation difficulties most reliably correlated with the lack of normal rightward lateralization of the P100 response to left ear temporally novel unattended sound (Table 4 Figure 9A). Correspondingly, when we contrasted P100 asymmetry indexes between subgroups of children with ASD who did or did not experience severe auditory abnormalities during early life, we found that the lack of normal rightward asymmetry characterized only the ‘sensory disturbed’ group (Figure 9B). This result is strikingly similar to what has been observed in our previous MEG study using binaural stimuli [34]. Interestingly, while only tendency for correlation between the atypical (relatively more leftward) P100 lateralization and IQ has been found in ASD in the previous MEG study, in the present EEG study the atypical P100 lateralization in the ASD participants significantly correlated with their developmental delay.

It is conceivable that the asymmetric reduction in highly affected individuals may reflect either functional hemispheric reorganization of deficient early orienting processes or pathological hyperexcitability in the left hemispheric auditory cortex. Interestingly, hemispheric rivalry and stronger hyperexcitability of the left hemisphere have been reported in subjects with left unilateral neglect and correlate with the severity of their left-sided extinction [130]. In line with this assumption, abnormally speeded saccades to the right-sided peripheral visual targets, that is, controlled by left hemispheric eye-fields, have been reported both in subjects with autism [131] and in patients with right brain damage and left-sided neglect [132].

Most importantly, the abnormal right-sided bias in ASD has also been found in the auditory domain [133]. Khalfa et al. [134] reported a strong abnormal right ear bias of the transiently evoked otoacoustic-emission (TEOAE) suppression effect in children with autism. Given that the TEOAE suppression effect is controlled by the contralateral efferent system including the medial olivocochlear pathways, which project directly onto the organ of Corti and are in turn modulated by the cortico-olivocochlear pathway originating in the auditory cortex, strong right ear predominance of TEAOE suppression is well-matched with our hypothesis on left hemispheric hyperexcitability in children with autism. It is conceivable that the suggested leftward bias of hemispheric excitability may underlie not only the abnormal asymmetry of the P100 response found in our study but also some behavioral and physiological asymmetries found in autism research.

To summarize, decreased P100 in the right hemisphere and its abnormal hemispheric lateralization in children with ASD may both result from similar developmental deficits in early preattentive arousal processes. The correlation between the P100 amplitude measures and behavioral abnormalities in ASD suggest that abnormal preattentive arousal in children with ASD is detrimental for their auditory behavior and may contribute to other behavioral abnormalities in ASD.

#### Comparison with previous studies that used the paired click paradigm

The present findings are generally in line with our earlier studies that applied the passive paired binaural click paradigm in children with autism or ASD. Similar to these previous EEG findings, the abnormalities in children with ASD were predominantly found for the right hemispheric obligatory CAEP components and for long ISIs (S1 click), pointing to a right-hemispheric deficit in processing temporally novel unattended sounds [31]. In good accordance with our prior MEG study of P100m components in older ASD children [34], the present study revealed reliable correlations of the P100 abnormal lateralization with auditory sensory modulation difficulties in ASD.

In spite of apparent similarities between our present EEG and the previous EEG and MEG findings, there are also some inconsistencies in the results that could be explained by variations in experimental paradigms, age, or clinical characteristics of particular samples.

Specifically, unlike the current results, the previous MEG study with children with ASD aged 8-14 years only revealed a tendency for P100m amplitude reduction in the right hemisphere and provided no evidence for different degrees of P100m abnormalities in response to S1 and S2 clicks. We think that this discrepancy is mainly explained by the different ages of participants in the two studies. As we discussed earlier, P100 can be most reliably investigated in younger children because of its gradual cancellation by N1b with age. The ASD-related P100 abnormality might therefore be more reliably revealed in the present EEG study, which included preschool and early school-age children, than in the previous MEG study where the majority of participants were over 10 years old.

Unexpectedly, unlike our previous EEG study of young children with autism that applied binaural clicks [28], the present study did not provide evidence for an abnormal N1c response to S1 in children with ASD. There may be at least one reason for this difference in results. The N1c abnormalities in ASD might be specifically related to *binaural* processing of temporally novel sound and could not be detected during *monaural* stimulation. In primates, for example, substantial differences between the cortical responses to monaural and binaural stimulation suggest that binaural interaction is an important contributing factor to the CAEP component amplitude [135]. To our knowledge, there are no studies that directly compare CAEPs in response to bi-vs monaural stimuli in children. However, judging by the available literature, mono- and binaural developmental CAEPs are strikingly different. For example, in children between 4 and 8 years of age, the N1c has been repeatedly described as the major component of CAEP to binaural sounds with long ISIs [28,49], while in the case of monaural stimulation, P100 dominates the response in the same latency range of 50-200 msec [57]. More studies are needed to clarify the effects of binaural interaction on CAEP in both typically developing children and those with ASD.

## Conclusion

In this study, we used pairs of unattended clicks presented to the left or right ear to examine preattentive arousal processes in typically developing children and children with autism spectrum disorders. We have found that a CAEP abnormality in processing a temporally novel S1 click in ASD is restricted by left ear stimulation. A strong dependency of the auditory P100 wave attenuation on the stimulated ear suggests right-lateralized abnormalities in the early preattentive modulatory influence on the auditory cortex. The lack of normal right hemisphere asymmetry in the P100 response to temporally novel clicks reliably correlates with the severity of early auditory-related behavioral abnormalities in ASD, including hyper- and hyposensitivity to sounds, confusion and aversive reactions to auditory stimulation. Although it must still be proven whether ASD individuals are characterized by a lateralized deficit in involuntary orienting toward unattended stimuli, similar to that found in patients with spatial neglect, this hypothesis is potentially attractive. Our findings suggest that some right-lateralized brain systems that are crucially important for arousal and attention re-orienting are compromised in individuals with autism.
